# Retraction Note: Inhibitory role of ATF3 in gastric cancer progression through regulating cell EMT and stemness

**DOI:** 10.1186/s12935-025-04066-5

**Published:** 2025-11-01

**Authors:** Chuanqian Huang, Renli Chen, Fangjing Zheng, Yirong Tang, Xiukang Wang, Zichun Chen, Xiaolan Lai

**Affiliations:** 1 Department of Medical Oncology and Radiotherapy, Ningde Municipal Hospital, Ningde Normal University, Ningde, 352000 Fujian China; 2 Department of Hematology and Rheumatism, Ningde Municipal Hospital, Ningde Normal University, Ningde, 352000 Fujian China; 3 Department of Pharmacy, Ningde Municipal Hospital Affiliated to Ningde Normal University, Ningde, 352000 Fujian China

**Retraction Note** **: Cancer Cell International (2021) 21:127**

10.1186/s12935-021-01828-9

The Editors-in-Chief have retracted this article. Following this article’s publication, concerns were raised regarding the similarity of Fig. 3d, MGC-803 cells (Control), with Fig. 3A, MGC-803 cells (siRNA-Ctrl) of [1] published by different authors. The authors failed to provide any explanation for the raised concerns.

The Editors-in-Chief therefore have lost confidence in this data.

None of the authors has responded to the Publisher regarding this retraction notice.
